# Supplementary material for: βPix Guanine Nucleotide Exchange Factor Regulates Regeneration of Injured Peripheral Axons
Source: Int J Mol Sci. 2023 Sep 20;24(18):14357. doi: 10.3390/ijms241814357 (PMC10532151; doi:10.3390/ijms241814357)
Supplement: Supplementary file 1 [file ijms-24-14357-s001.zip › ijms-2557727-supplementary.pdf]

Supplementary Materials

βPix guanine nucleotide exchange factor regulates regeneration of injured peripheral axons

Yewon Jeon, Yoon Kyung Shin, Hwigyeong Kim, Yun Young Choi, Minjae Kang, Younghee Kwon, Yongcheol Cho, Sung Wook Chi, and Jung Eun Shin

Contents: Three supplementary figures

Figure S1: Axon regeneration one day after sciatic nerve crush injury, related to Figure 3F-3H
Figure S2: Figure S2. Phosphorylated Src levels after sciatic nerve crush injury, related to Figure 5
Figure S3: Original images for western blots

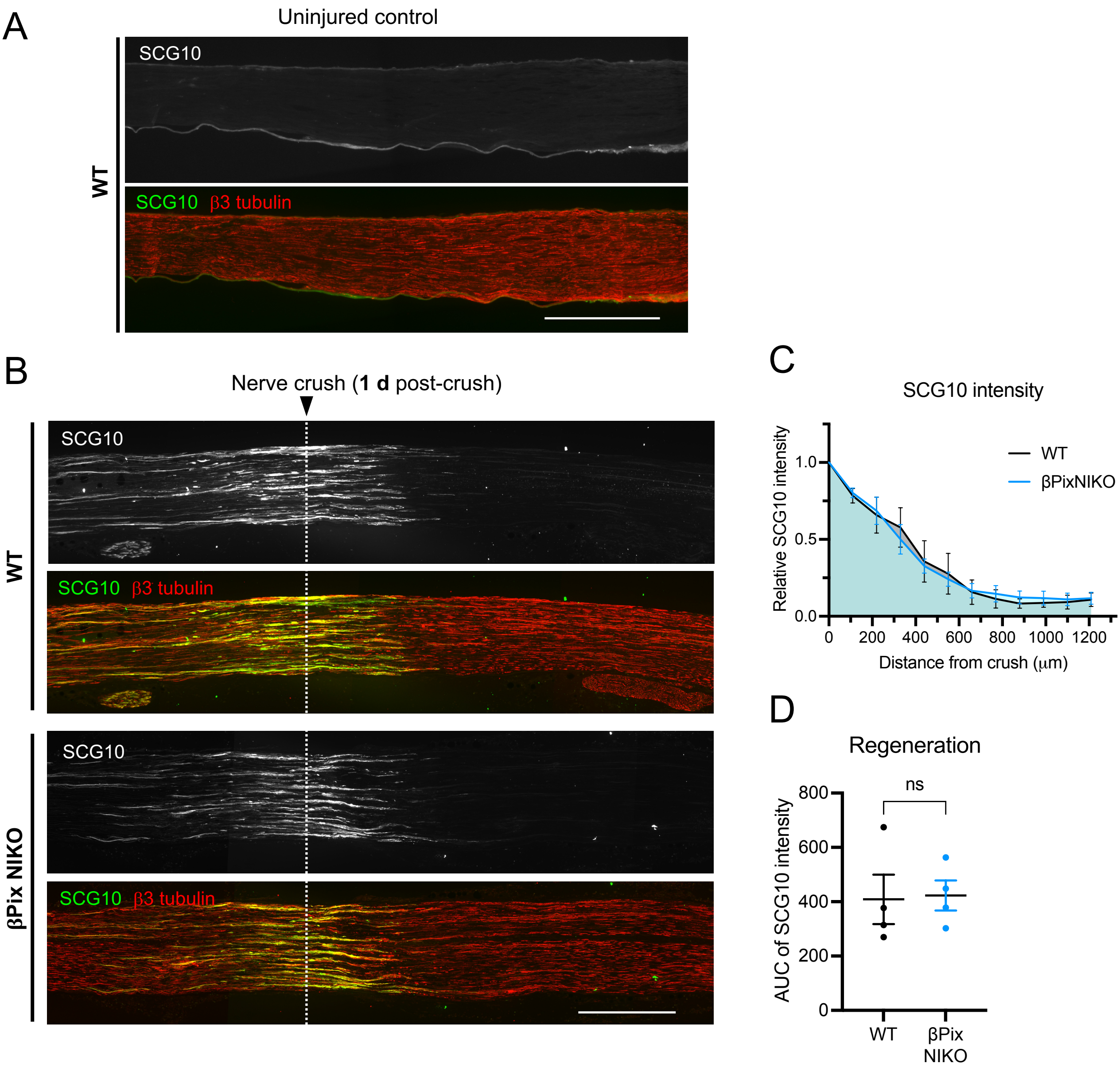

Figure S1. Axon regeneration one day after sciatic nerve crush injury, related to Figure 3F-3H

A. Longitudinal section of uninjured wild-type (WT) mouse sciatic nerve, stained for SCG10 (a regenerating axonal marker) and β3 tubulin. The basal SCG10 level is very low. Scale bar, 500 μm.
B. Longitudinal sections of WT and βPix neuronal isoform knockout (NIKO) mouse sciatic nerves one day after crush injury, stained for SCG10 and β3 tubulin. SCG10 immunoreactivity reveals axonal regeneration up to approximately 500 μm-distal to the injury site in both genotypes. Scale bar, 500 μm.
C. Data shown in (**B**) were subjected to intensity analysis for SCG10. SCG10 intensity was not significantly different between the genotypes (n = 4).
D. Area under curve (AUC) values of SCG10 intensity plots shown in (**C**) were not significantly different between the genotypes (n = 4).
Error bars represent mean ± SEM.

Figure S1, Jeon et al.

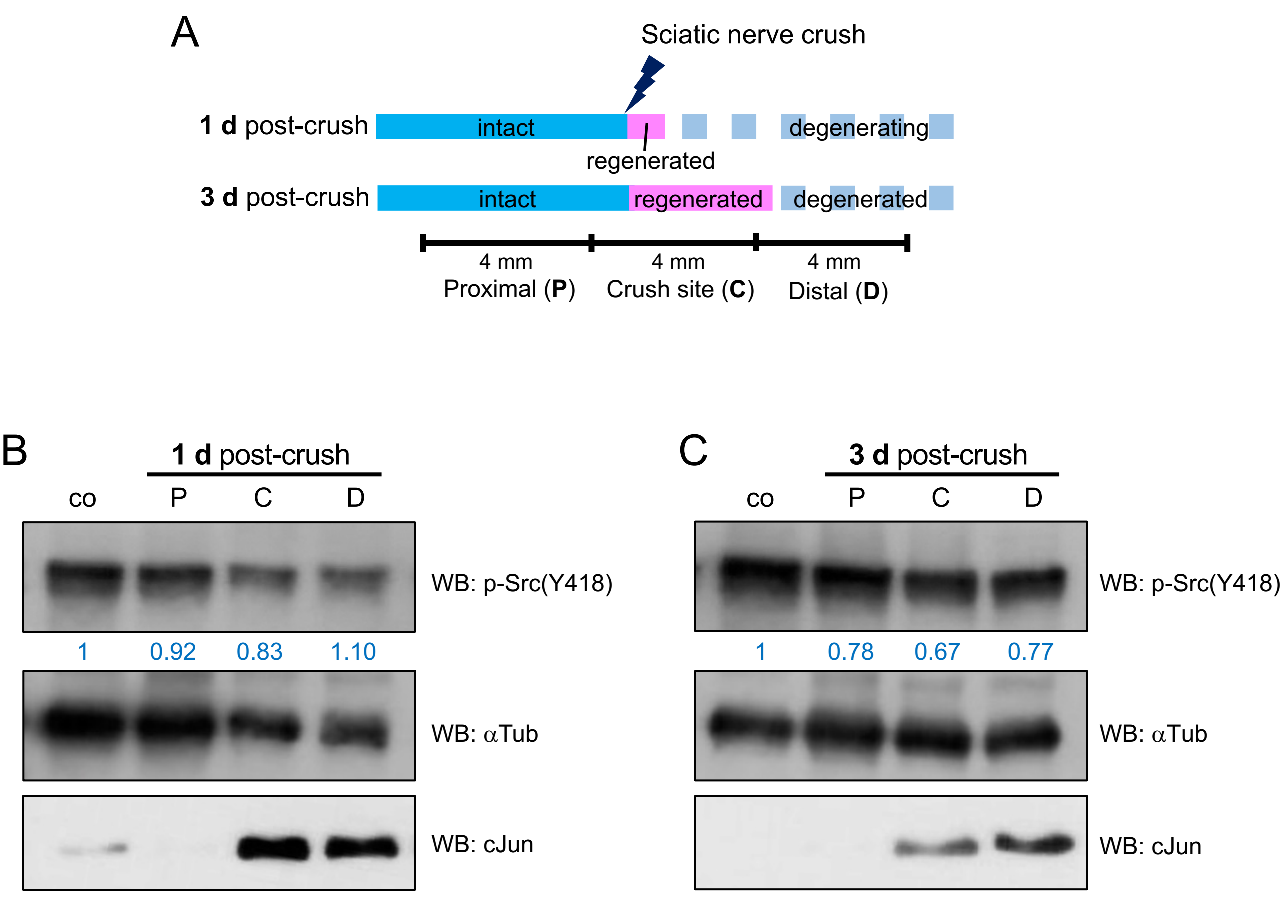

Figure S2. Phosphorylated Src levels after sciatic nerve crush injury, related to Figure 5

A. Schematic diagram of crushed sciatic nerve, depicting proximal (P), crush site (C), and distal (D) segments. Pink box indicates the region with regenerating axons at one or three days after injury.

B-C. Western blot results showing phosphorylated Src (p-Src Y418) levels in indicated nerve segments one day (panel **B**) or three days (panel **C**) after crush. p-Src Y418 levels are shown as relative values to uninjured control nerve (co) after normalization to the loading control, αTub. cJun upregulation in the crush sites and distal segments is used as a marker of nerve injury.

Figure S2, Jeon et al.

Raw images used in Figure 1D

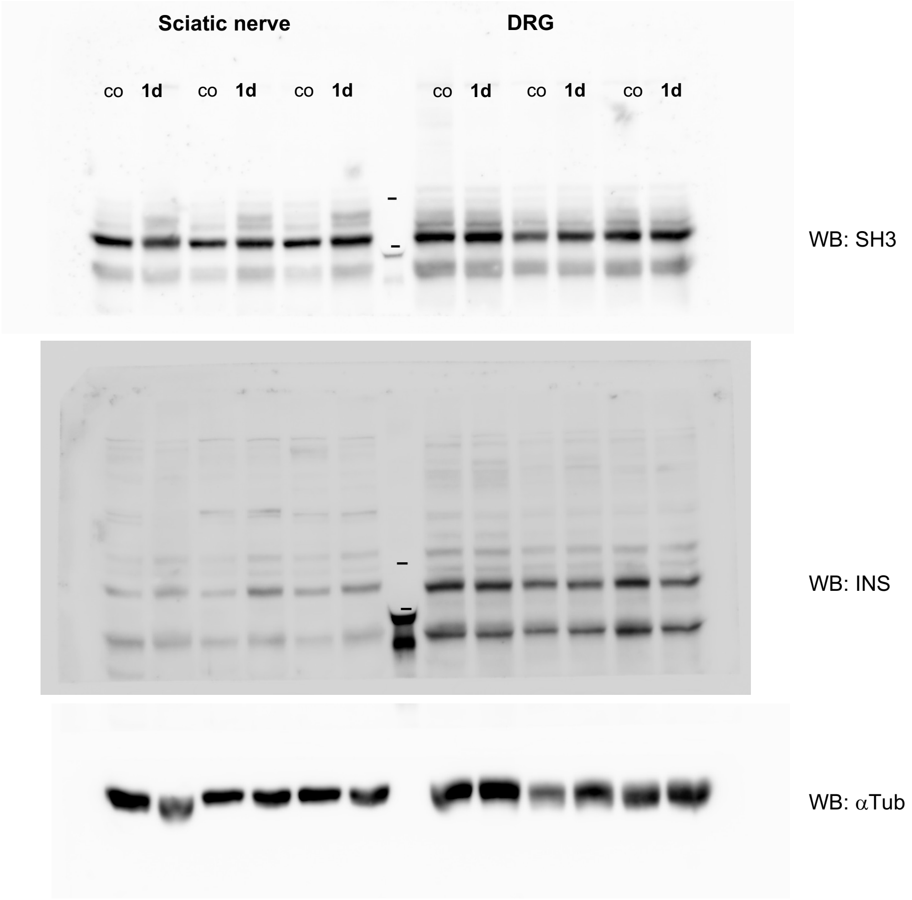

Figure S3A, Jeon et al.

Raw images used in Figure 1F

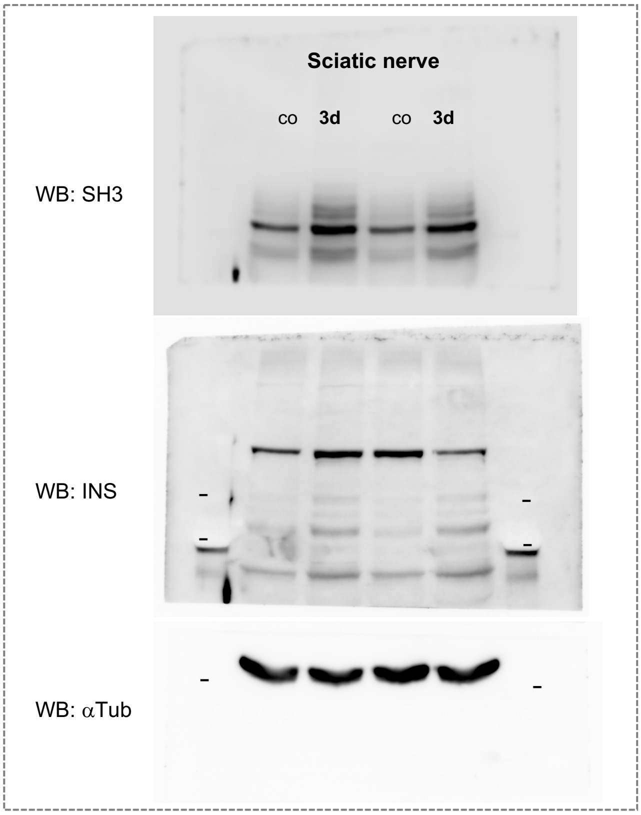

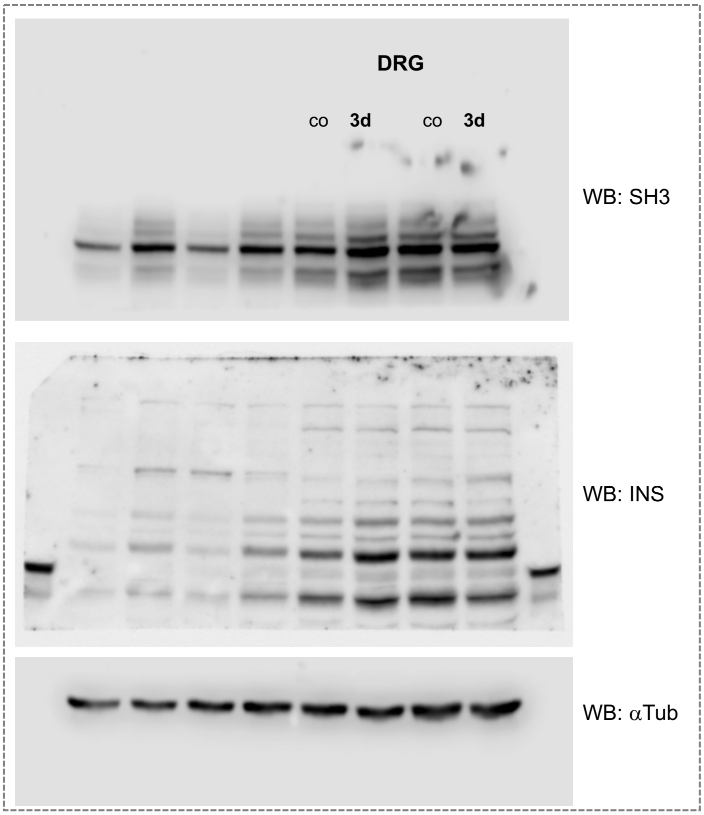

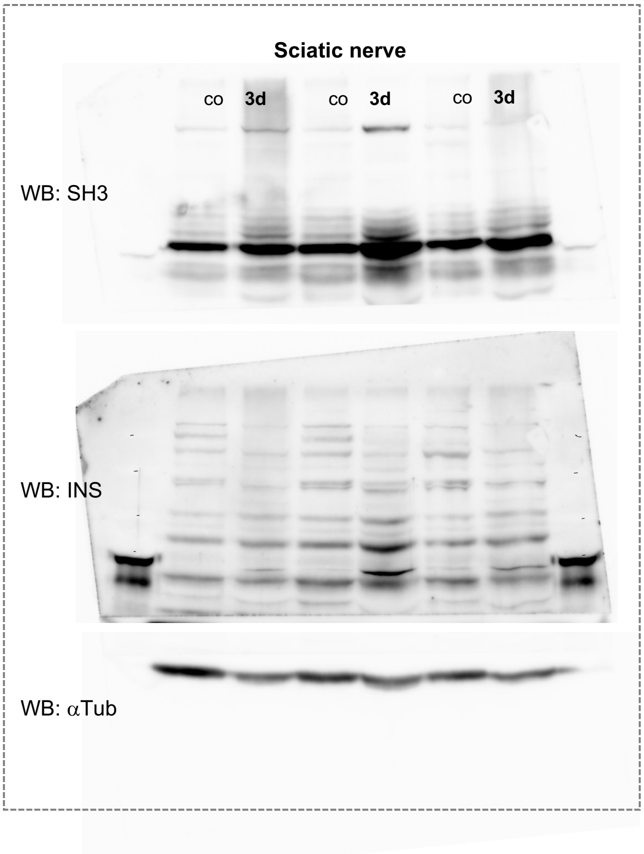

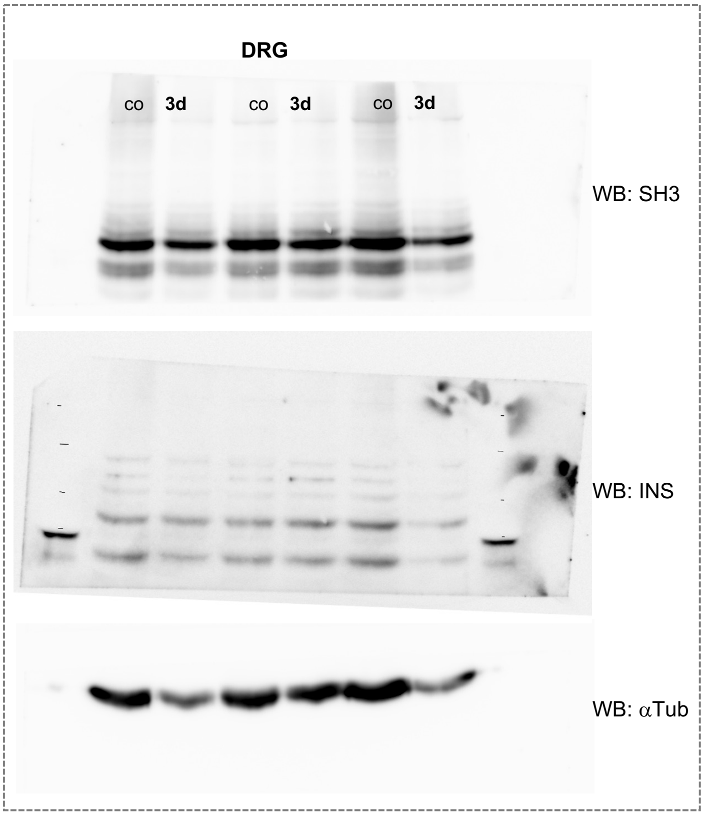

Figure S3B, Jeon et al.

Raw images used in Figure 1H

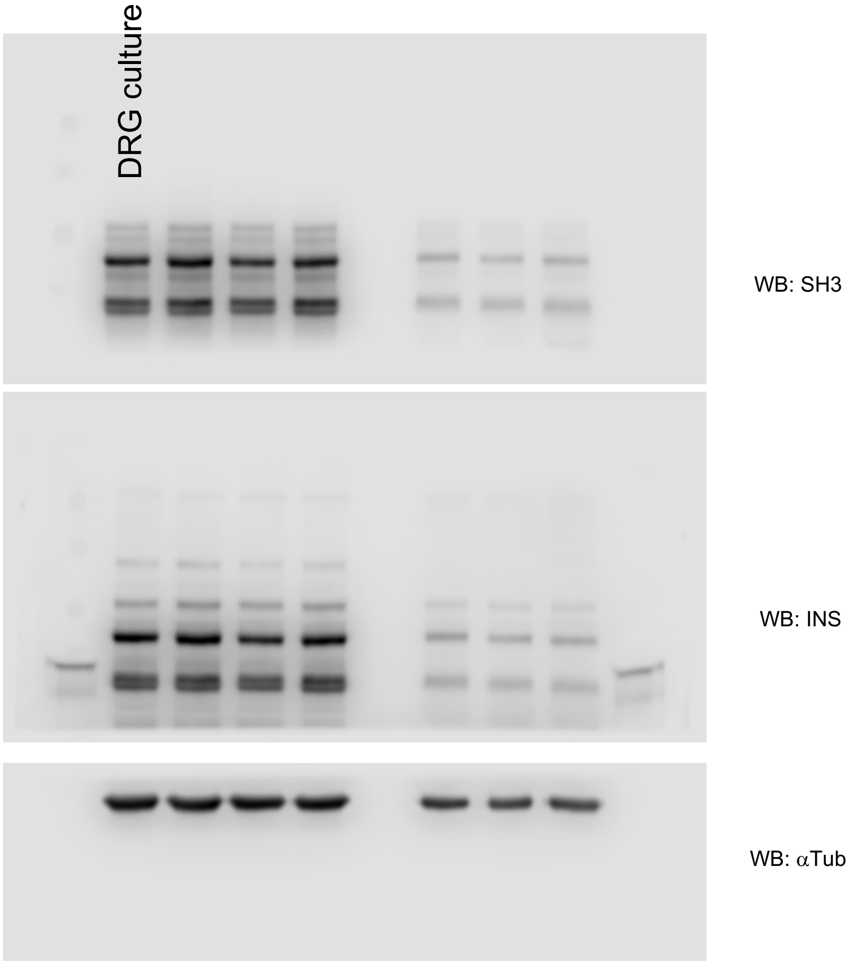

Figure S3C, Jeon et al.

Raw images used in Figure 1I

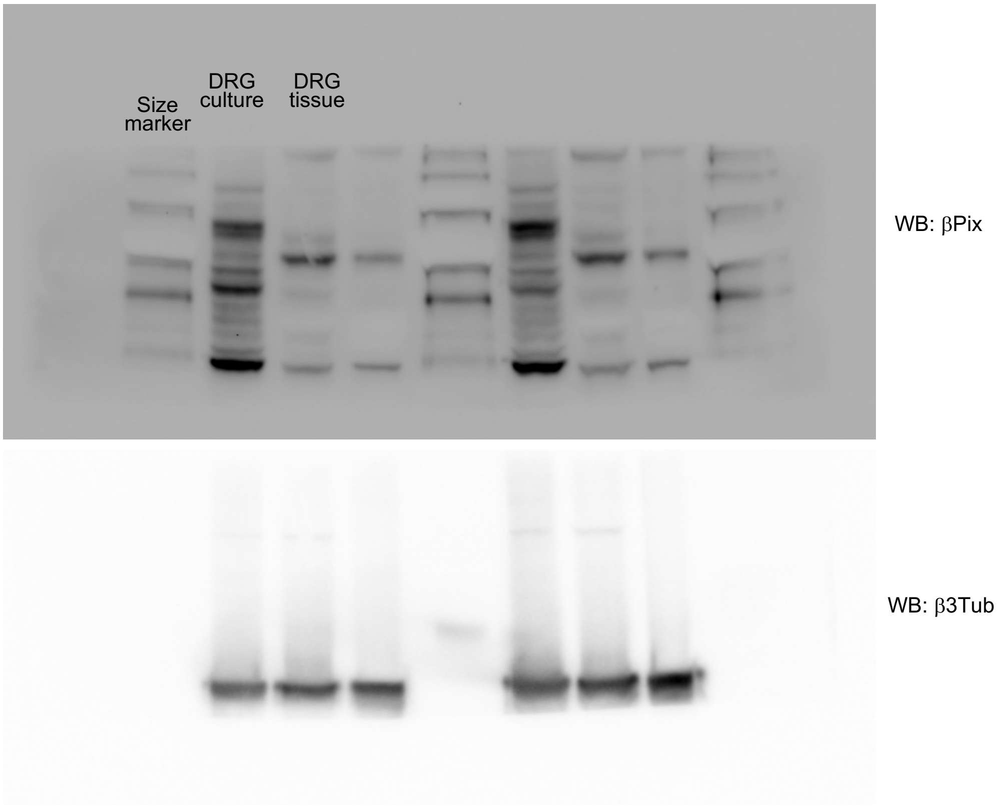

Figure S3D, Jeon et al.

Raw images used in Figure 3A

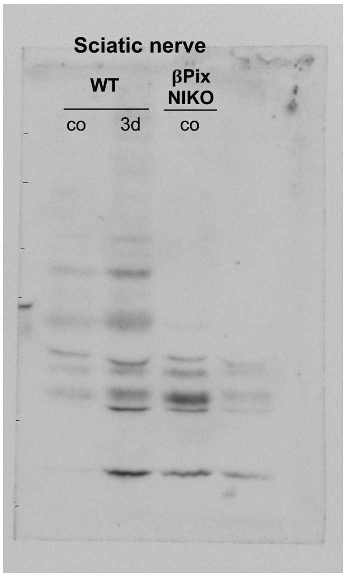

WB: INS

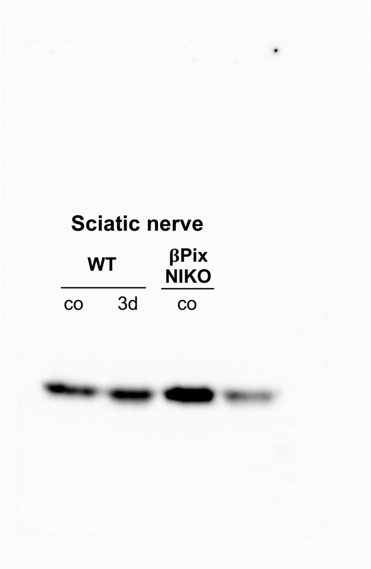

WB: αTub

Figure S3E, Jeon et al.

Raw images used in Figure 4A

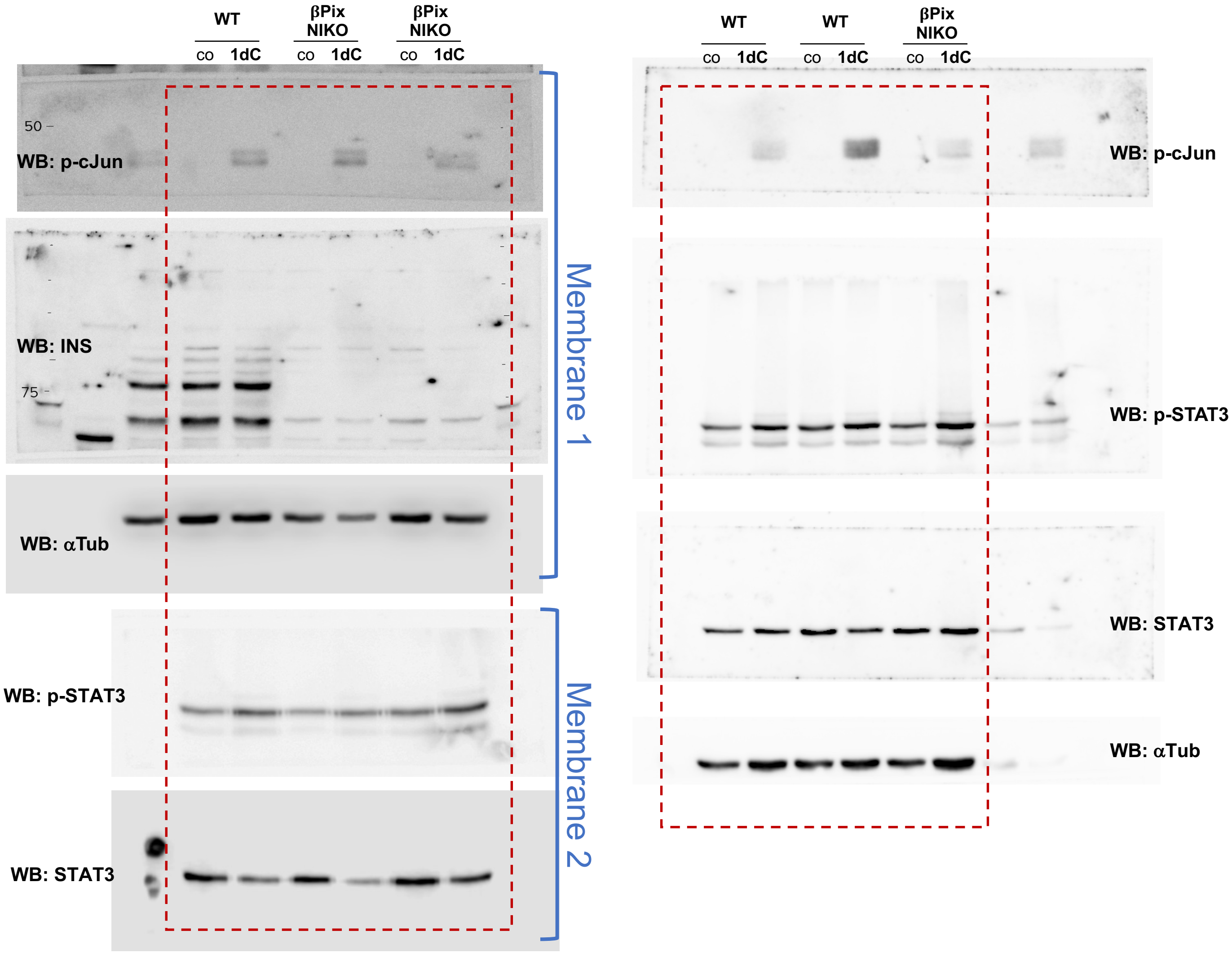

The dotted red boxes indicate the data used for quantification. Other lanes were not used because the loading control levels were abnormally low.

Figure S3F, Jeon et al.
